# Supplementary material for: Functional Exploitation of Carob, Oat Flour, and Whey Permeate as Substrates for a Novel Kefir-Like Fermented Beverage: An Optimized Formulation
Source: Foods. 2021 Feb 1;10(2):294. doi: 10.3390/foods10020294 (PMC7912806; doi:10.3390/foods10020294)

Figure S1. Separation by HPLC (280 nm) of microbial derivatives of phenolic acids in methanol/water-soluble extracts obtained from the unfermented mixture (A) and fermented KLB (B).

A

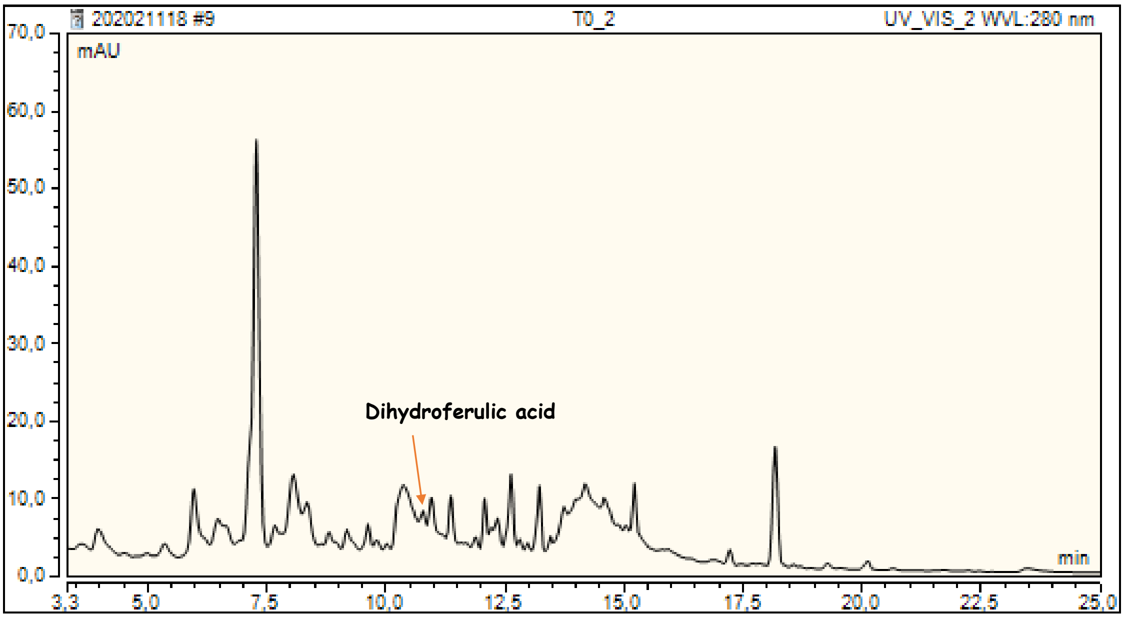

B

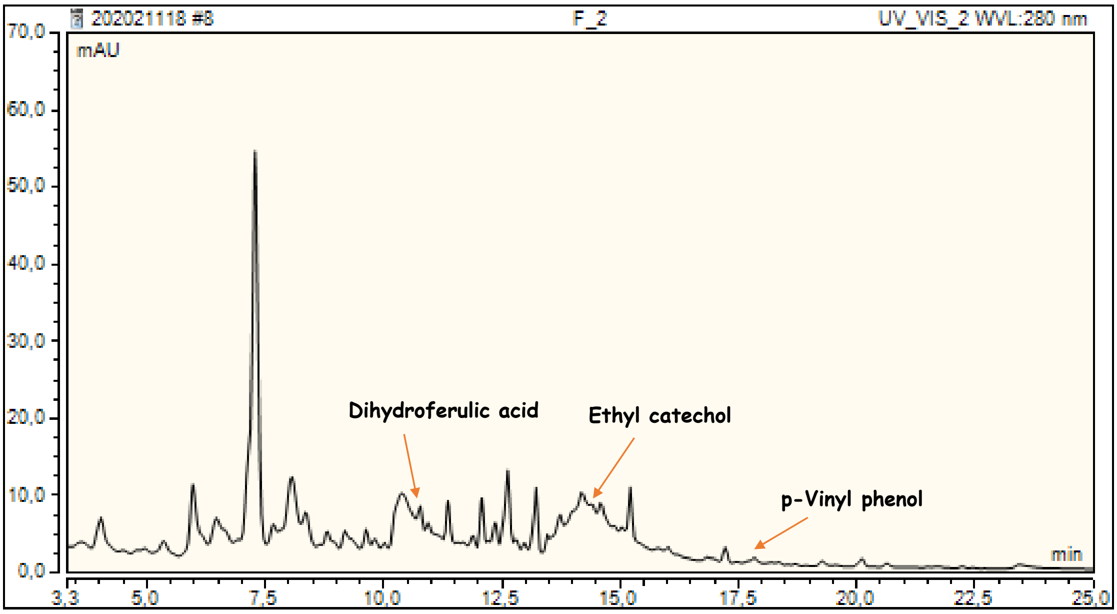

Supplement: Supplementary file 1 [file foods-10-00294-s001.zip › Figure S1.pdf]
