# Supplementary material for: Functional Exploitation of Carob, Oat Flour, and Whey Permeate as Substrates for a Novel Kefir-Like Fermented Beverage: An Optimized Formulation
Source: Foods. 2021 Feb 1;10(2):294. doi: 10.3390/foods10020294 (PMC7912806; doi:10.3390/foods10020294)
Supplement: Supplementary file 1 [file foods-10-00294-s001.zip › Table S1.pdf]

Table S1. Mass transitions (MRM) and instrumental parameters optimized for phenolic compounds analyzed by LC-ESI-MS/MS.

Compound	Ionization Mode	Precursor ion	Q1		Q2		Q2		RT (min)
			Product ion Q1	Collision energy (V)	Product ion Q2	Collision energy (V)	Product ion Q2	Collision energy (V)	
Chlorogenic acid	-	353	191	20					5.21
Catechin	-	289	203	20	123	32			5.36
Caffeic acid	-	179.1	135.1	17	134.1	28	89.2	44	5.54
p-coumaric acid	+	165.1	119.2	16	91.2	25	65.3	33	6.31
Vanillin	+	153	125	10	93	16			6.34
Hyperoside	-	463.2	300.1	26	271.1	21			6.87
Ferulic acid	+	195	145	50	117	24			6.93
Isoquercetin	-	463.1	300	42	270.9	26			6.97
Luteolin	+	287	153	32	135	30			9.31
