# Supplementary material for: Functional Exploitation of Carob, Oat Flour, and Whey Permeate as Substrates for a Novel Kefir-Like Fermented Beverage: An Optimized Formulation
Source: Foods. 2021 Feb 1;10(2):294. doi: 10.3390/foods10020294 (PMC7912806; doi:10.3390/foods10020294)
Supplement: Supplementary file 1 [file foods-10-00294-s001.zip › Table S2.pdf]

Table S2. Analysis of variance for each response (lactic acid bacteria and yeasts cell counts, pH, total titratable acidity [TTA], total phenolics content [TPC], DPPH radical scavenging activity, and overall acceptability [OA]).

Source	Sum of squares	DF	Mean squares	F-value	Signif.%
**Lactic acid bacteria cells count log (CFU/ml)**					
**Model**	3.1541	5	0.6308	95.6258	***
**Residual**	0.0462	7	0.0066		
**Lack of fit**	0.0218	3	0.0073	1.1900	42.0%
**Pure error**	0.0244	4	0.0061		
**total**	3.2002	12			
R^2= 0.986 ; Adj-R^2 =0.975					

(* $p < 0.05$, ** $p < 0.01$, and *** $p < 0.001$).

Source	Sum of squares	DF	Mean squares	F-value	Signif.%
**Yeasts cells count (log CFU/ml)**					
**Model**	5.18015E-0001	5	1.03603E-0001	6598.9158	***
**Residual**	4.17591E-0002	7	5.96559E-0003		
**Lack of fit**	4.16963E-0002	3	1.38987E-0002	885.2720	***
**Pure error**	6.28000E-0005	4	1.57000E-0005		
**total**	5.59774E-0001	12			
R^2=0.925 ; Adj-R^2 =0.872					

(* $p < 0.05$, ** $p < 0.01$, and *** $p < 0.001$).

Source	Sum of squares	DF	Mean squares	F-value	Signif.%
**pH**					
**Model**	0.0349	5	0.0070	58.0935	**
**Residual**	0.0056	7	0.0008		
**Lack of fit**	0.0051	3	0.0017	14.2800	*
**Pure error**	0.0005	4	0.0001		
**total**	0.0405	12			
R^2= 0.861; Adj-R^2 =0.762					

(* $p < 0.05$, ** $p < 0.01$, and *** $p < 0.001$).

Source	Sum of squares	DF	Mean squares	F-value	Signif.%
**TTA**					
**Model**	0.0878	5	0.0176	219.3988	***
**Residual**	0.0077	7	0.0011		
**Lack of fit**	0.0073	3	0.0024	30.6142	**
**Pure error**	0.0003	4	0.0001		
**total**	0.0954	12			
R^2= 0.920; Adj-R2 =0.862					

(* $p < 0.05$, ** $p < 0.01$, and *** $p < 0.001$).

Source	Sum of squares	DF	Mean squares	F-value	Signif.%
**TPC (mg GAE/mL)**					
**Model**	230.2032	5	46.0406	580.3687	***
**Residual**	45.4356	7	6.4908		
**Lack of fit**	45.1183	3	15.0394	189.5807	***
**Pure error**	0.3173	4	0.0793		
**total**	275.6389	12			
R^2= 0.835; Adj-R2 =0.717					

(* $p < 0.05$, ** $p < 0.01$, and *** $p < 0.001$).

Source	Sum of squares	DF	Mean squares	F-value	Signif.%
**DPPH radical scavenging activity (%)**					
**Model**	210.5275	5	42.1055	57.6211	**
**Residual**	47.5512	7	6.7930		
**Lack of fit**	44.6283	3	14.8761	20.3579	**
**Pure error**	2.9229	4	0.7307		
**total**	258.0787	12			
R^2= 0.816 ; Adj-R2 =0.684					

(∗p < 0.05, ∗∗p < 0.01, and ∗∗∗p < 0.001).

Source	Sum of squares	DF	Mean squares	F-value	Signif.%
**OA (5 point hedonic scale)**					
**Model**	0.6039	5	0.1208	38.8140	***
**Residual**	0.0218	7	0.0031		
**Lack of fit**	0.0069	3	0.0023	0.6132	64.3%
**Pure error**	0.0149	4	0.0037		
**total**	0.6257	12			
R^2= 0.965; Adj-R^2 =0.940					

(∗p < 0.05, ∗∗p < 0.01, and ∗∗∗p < 0.001).
